# Heart–Brain Axis: A Narrative Review of the Interaction between Depression and Arrhythmia

**DOI:** 10.3390/biomedicines12081719

**Published:** 2024-08-01

**Authors:** Shuping Fang, Wei Zhang

**Affiliations:** 1 Mental Health Center of West China Hospital, Sichuan University, Chengdu 610041, China; shupingfangdr@163.com; 2 West China Biomedical Big Data Center, West China Hospital, Sichuan University, Chengdu 610041, China

**Keywords:** depression, arrhythmia, autonomic nervous system, heart, brain, interoception

## Abstract

Arrhythmias and depression are recognized as diseases of the heart and brain, respectively, and both are major health threats that often co-occur with a bidirectional causal relationship. The autonomic nervous system (ANS) serves as a crucial component of the heart–brain axis (HBA) and the pathway of interoception. Cardiac activity can influence emotional states through ascending interoceptive pathways, while psychological stress can precipitate arrhythmias via the ANS. However, the HBA and interoception frameworks are often considered overly broad, and the precise mechanisms underlying the bidirectional relationship between depression and arrhythmias remain unclear. This narrative review aims to synthesize the existing literature, focusing on the pathological mechanisms of the ANS in depression and arrhythmia while integrating other potential mechanisms to detail heart–brain interactions. In the bidirectional communication between the heart and brain, we emphasize considering various internal factors such as genes, personality traits, stress, the endocrine system, inflammation, 5-hydroxytryptamine, and behavioral factors. Current research employs multidisciplinary knowledge to elucidate heart–brain relationships, and a deeper understanding of these interactions can help optimize clinical treatment strategies. From a broader perspective, this study emphasizes the importance of considering the body as a complex, interconnected system rather than treating organs in isolation. Investigating heart–brain interactions enhance our understanding of disease pathogenesis and advances medical science, ultimately improving human quality of life.

## 1. Introduction

Depression is a mood disorder characterized by a persistent low mood or loss of interest [1]. It is prevalent, with approximately one in five individuals experiencing it at least once in their lifetime [2]. Additionally, over 700,000 individuals succumb to suicide annually due to depression [3]. By 2030, depression is projected to become the leading cause of global disease burden [2]. Cardiovascular disease (CVD) encompasses various conditions affecting the heart and circulatory system, ranking as one of the leading causes of mortality worldwide [4,5]. There is a robust association between depression and physical ailments such as CVD [1]. Studies indicate that depression increases the risk of cardiovascular events and significantly influences the severity of CVD [6,7]. Conversely, CVD can also trigger depressive symptoms [8]. The simultaneous occurrence of CVD and psychological disorders is referred to as psycho-cardiological diseases [9].

One of the primary manifestations of CVD is arrhythmia, characterized by irregular heart rhythms [10]. Arrhythmias contribute significantly to global morbidity and mortality rates [11]. The development of arrhythmias is primarily influenced by myocardial electrical instability and both acute and chronic psychological stress [12]. Atrial fibrillation (AF) and atrial flutter are the most prevalent subtypes of arrhythmia [13]. There is a bidirectional relationship between depression and arrhythmia, with negative emotions potentially triggering or exacerbating CVD symptoms [9,14,15]. Depression is associated with an increased incidence of adverse cardiovascular events [9] and emotional stress is a well-recognized trigger for ventricular arrhythmias and sudden cardiac death [16]. Major depressive disorder (MDD) and its severity are independent predictors of fragmented QRS and the Tp-Te/QT ratio [17,18], and depression is also closely associated with non-ventricular arrhythmias. Patients with symptomatic AF have shown improvements in anxiety and depression symptoms following catheter ablation [19], whereas poor mental health may increase the likelihood of AF recurrence post-ablation [20]. A meta-analysis of seven cohort studies involving 1070 AF patients found that depression independently raises the risk of AF recurrence post-ablation [21]. About 38% of AF patients exhibit depressive symptoms [22], and around 25% of individuals with depression or depressive symptoms have AF [23]. Despite the high incidence rates of psycho-cardiological disorders, clinical diagnosis rates remain low [9,24]. Currently, there is still a lack of definitive treatment for the co-occurrence of arrhythmia and depression [24]. Antiarrhythmic and antidepressant medications can have side effects, and non-pharmacological treatments, including psychotherapy, are being explored by researchers [9,25,26].

Although the interaction mechanisms between depression and arrhythmia remain unclear, the heart–brain axis (HBA) theory may offer valuable insights into their inter-relationship. The HBA describes the intricate and reciprocal connection between the heart and brain, influencing mood disorders and CVDs [27]. The elucidation of heart–brain interactions has been a prolonged journey. Historically, the brain has been viewed as the “commander-in-chief” of the body, with physiological states and brain functions considered separately [28,29]. However, research using techniques like positron emission tomography (PET) and electroencephalography (EEG) has shown dynamic heart–brain coupling. PET studies revealed that myocardial ischemia activates the thalamus [30], while EEG studies demonstrated that heartbeat-evoked potentials (HEP) correlate with stress-induced changes in cardiac output and HEP amplitude variations, indicating heart–brain interaction [16,31]. In recent decades, further investigations have elucidated the mechanisms underlying this interaction. Although the heart and brain function independently as key organs of the circulatory and central nervous systems (CNS), they are anatomically interconnected through the autonomic nervous system (ANS) [27]. The nervous system plays a crucial role in cardiovascular physiology and pathophysiology [32]. Psychological processes and their neural substrates are intimately connected to the regulation of internal bodily states [28]. Over the past decade, research on interoception has surged sixfold [33]. Interoception refers to the perception of internal bodily states and processes, wherein peripheral visceral information is relayed to the brain, exerting both direct and indirect influences on brain function [28]. Interoception, based on the ANS, is vital for physiological regulation, emotional experience, and behavioral modulation [34]. Nonetheless, autonomic neurology alone does not suffice to fully elucidate arrhythmias and depressive disorders; these pathways are influenced by external stress, endocrine, immune, and behavioral factors, as well as genes and neurotransmitters at the molecular level [27,35].

In summary, there is a significant bidirectional causal relationship between depression and arrhythmia [9], and this relationship suggests an interaction within the HBA to some extent [27]. However, the mechanisms underlying this interaction are not yet fully understood, and effective clinical treatments for these conditions are lacking [25,26]. An in-depth investigation of the interaction mechanisms between arrhythmia and depressive symptoms can enhance our understanding of mood disorders and cardiac diseases, thereby enriching knowledge of heart–brain interactions. A comprehensive elucidation of these interactive mechanisms can provide crucial insights for clinical treatments, addressing these major health threats. We propose that the ANS plays a pivotal role in the pathogenesis of both depression and arrhythmia, interacting with other potential pathological mechanisms.

This narrative review aims to synthesize the existing literature, focusing on the pathological mechanisms of the ANS in depression and arrhythmia while integrating other potential mechanisms within heart–brain interactions. Additionally, it reviews the current state of research and outlines future directions, providing essential information and new perspectives for the study of heart–brain interactions.

## 2. The Interoceptive Pathway of Heart–Brain Interactions

In the unconscious state, the heart can autonomously generate intrinsic electrical activity and maintain its rhythm even when disconnected from the brain. The cardiac cycle is regulated by descending influences from the CNS and local influences from the heart’s intrinsic nervous system [36]. Simultaneously, continuous information is conveyed from the heart to the brain via an ascending interoceptive pathway, where it is perceived and processed [34,36].

### 2.1. Neuroanatomical Basis

**Figure 1:**
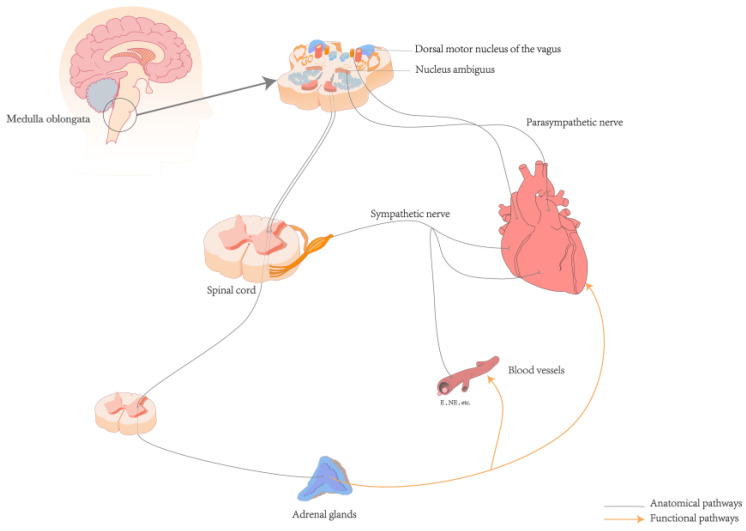
Anatomical and functional pathways of the heart and brain. Abbreviations: E: Epinephrine; NE: Norepinephrine.

The neuroanatomical pathway from the heart to the brain primarily involves a complex network of the autonomic and CNS [35]. The ANS, comprising the sympathetic and parasympathetic nerves, is interconnected with surrounding organs and plays a regulatory role in various systems such as the cardiovascular, respiratory, digestive, and endocrine systems [37]. Sympathetic afferent fibers carry signals from baroreceptors and chemoreceptors in the heart through the cardiac plexus to the sympathetic chain ganglia, entering the spinal cord via thoracic spinal nerves (T1–T5). These fibers first pass through the dorsal root ganglia, which are located on the dorsal roots just outside the spinal cord. The fibers then proceed through the dorsal roots into the spinal cord, ultimately reaching the dorsal horn where they synapse with secondary neurons. The signals are then propagated along the spinal cord to the nucleus tractus solitarius (NTS) in the medulla oblongata [35,38]. Afferent signals from sensory receptors in the heart are transmitted via the afferent fibers of the vagus nerve, the tenth cranial nerve, which is a major component of the parasympathetic nervous system. These signals arise from the intrinsic plexuses within the cardiac branches of the vagus nerve, travel through the main trunk of the vagus nerve, and ascend through the thoracic cavity into the cervical region. In the cervical region, the signals pass through the vagal ganglia, including the jugular ganglion and the nodose ganglion, before continuing upward into the cranial cavity. Upon reaching the medulla oblongata, these afferent fibers enter the dorsal motor nucleus of the vagus and the nucleus ambiguus, both of which are critical parasympathetic nuclei, where they integrate and relay signals. Finally, the fibers terminate at the NTS [38,39]. The NTS acts as a relay station for the interoceptive ascending pathway [38,39]. Within the NTS, sensory information is integrated and processed before being transmitted to other brainstem nuclei and the cerebral cortex, facilitating regulation at both the ANS level and the level of conscious perception [35] (See Figure 1).

The central autonomic network (CAN) is a complex neural network distributed throughout the brain, including the insular cortex, amygdala, hypothalamus, periaqueductal gray matter, parabrachial complex, NTS, ventrolateral medulla, and other regions [40]. The CAN is intricately linked with both the sympathetic and parasympathetic nervous systems. Under stress conditions, the CAN enhances sympathetic nervous activity, leading to the release of norepinephrine, which interacts with beta-1 adrenergic receptors on myocardial cells. This results in increased cardiac contractility, elevated heart rate, increased blood pressure, and enhanced blood supply [41,42]. Conversely, under relaxed conditions, the CAN enhances parasympathetic activity, resulting in the release of acetylcholine, which acts on M-type cholinergic receptors on myocardial cells, reducing heart rate and promoting digestion and metabolism [41,42]. Through complex neural connections and feedback mechanisms, the CAN integrates and regulates the ANS, emotional states, and stress responses, maintaining homeostasis to ensure the body’s balance and adaptive responses across different physiological and behavioral states. The CAN is vital for the downward regulation of internal movement, neuroendocrine function, and survival-related behavioral responses [40]. Currently, experimental research primarily focuses on the descending influences from the brain to the heart [36], such as the modulation of heart rate and heart rate variability(HRV) by cortical brain regions [34,36,43].

### 2.2. Ascending Pathway

Interoception refers to an individual’s perception of their own internal physiological state, including sensations such as heartbeat, breathing, hunger, and thirst [28]. It provides crucial information about the body’s state and plays a key role in emotion generation and regulation [33]. The ANS acts as a vital relay between the viscera and the brain, forming the anatomical basis of interoception [37,38,44]. A comprehensive understanding of the anatomy and physiology of this pathway is essential for elucidating heart–brain interactions [33]. The ascending pathway of interoception originates in internal organs and peripheral receptors detecting chemical, mechanical, or temperature changes [33]. Peripheral information is transmitted via cranial nerves and the ANS to the NTS, a primary visceral receiving area in the brainstem. From the NTS, signals are further transmitted to higher-level cortical and subcortical structures, including the parabrachial nucleus (PBN), thalamus, hypothalamus, hippocampus, amygdala, insular cortex, anterior cingulate cortex and somatosensory cortex [35]. This pathway influences emotional processing and responses to some extent [33,35]. Interoception helps regulate homeostasis and maintain organismal survival [34]. Part of the interoceptive signals is modulated by descending control from the CNS over the ANS, forming an interactive loop that regulates bodily organs and a wide range of motor, cognitive, and emotional processes.

The ascending pathway of interoception is a complex and multi-system process. Humoral receptor regulation can interact with the autonomic nerves to regulate cardiac activity. For example, high levels of catecholamine secretion in the sympathetic-adrenal medullary system can increase myocardial excitability and cortisol levels, thereby promoting the occurrence of arrhythmias (See Figure 1) [6,45]. The adrenal glands, which are regulated by spinal central nervous control, release cortisol under the regulation of the hypothalamic-pituitary-adrenal (HPA) axis [46]. Elevated cortisol levels are significantly associated with more persistent depressive symptoms [47]. Baroreceptors in the aortic arch and carotid sinus detect mechanical changes in blood vessel walls caused by blood flow variations. This afferent information is transmitted via glossopharyngeal and vagus nerves to the NTS, which plays a role in perception, cognition, and behavior. Efferent signals from NTS, orbitofrontal cortices, thalamus, insular cortex, and other brain regions involved in its structure and function act upon the cardiovascular system to regulate blood pressure fluctuations. These findings suggest dynamic coupling between cardiovascular activity at a cerebral level [16,28,30,31,48]. The ANS receives ascending information from peripheral mechanoreceptors, chemoreceptors, osmoreceptors along with other pathways. The collaborative interplay between neuromodulation and interoceptive sensors establishes an effective communication bridge that connects the heart and brain [34,35,36]. This interaction forms the basis for the dynamic interplay influencing perception, cognition, emotion, and physical health [12,36,49].

## 3. The Involvement of the ANS in the Pathogenesis of Arrhythmias Triggered by Mental Stress

Autonomic regulation is vital for homeostasis [50], and closely interconnects cardiac and brain activities, facilitating the body’s adaptation to dynamic external environments [51]. Stress can affect the ANS through various mechanisms [50]. Firstly, mental stress can directly impact ANS activity by releasing neurotransmitters and altering neuron excitability, leading to sympathetic overactivity and parasympathetic inhibition, thereby increasing the risk of arrhythmia [52]. Prolonged chronic stress can also lead to pathological changes in the ANS. Secondly, mental stress can indirectly influence the ANS by affecting the HPA axis, which is closely related to stress regulation. Mental stress may cause HPA axis hyperactivity, subsequently impacting ANS function. Additionally, mental pressure has the potential to modulate ANS function through its effects on brain structure and function.

The pathophysiology of arrhythmia remains incompletely understood; however, aberrant activation of the sympathetic nervous system (SNS) is a key etiological factor [52]. Excessive SNS activation can be triggered by mental stress [50], inducing cardiac electrical instability and abnormal excitation, and impaired conduction within cardiac cells. Pathological processes such as psychological stress, ischemia, myocardial infarction, and cellular and neural remodeling can exacerbate sympathetic activation and exaggerated β-adrenergic signaling. Consequently, ventricular action potential duration (APD) and refractory periods are shortened, thereby establishing a detrimental cycle perpetuating arrhythmic events [16,53].

Changes in the ANS contribute to arrhythmia and disruptions in bodily functions [52], and are implicated in various mood disorders, including bipolar disorder, depression, and anxiety [54,55,56,57]. The anatomical structure of the ANS within the HBA underscores its pivotal role in linking CVD such as arrhythmia with emotional disorders [54,56]. Recent studies have revealed that both depression and anxiety are associated with elevated heart rate and reduced HRV [57,58], even in individuals with mood disorders who do not use psychiatric medications [59]. Abnormal cardiac activity and dysregulation of the ANS are considered consequences of psychological disorders through descending regulation mechanisms [21,58,60,61,62], and are also recognized as a risk factor for CVD in mental illnesses like depression [59].

Parasympathetic nerves may exert antiarrhythmic effects, such as the prevention of ventricular fibrillation during acute ischemia in dogs through direct electrical stimulation of the right vagus nerve, confirming the antifibrillation effect of vagal activation [52]. Additionally, parasympathetic nerves appear to have a protective influence on mood regulation. Negative emotions are associated with reduced parasympathetic activity and increased arrhythmias [54,60], while positive emotions may enhance parasympathetic activity [63,64]. Specifically, an elevated sympathetic nerve activity can potentially heighten individual susceptibility to stress [65,66], and depression severity is positively correlated with heart rate and negatively correlated with HRV [58,62]. These findings also suggest that patients with depression exhibit diminished ANS function and impaired cardiac adaptability [66,67].

However, gender differences exist in the ANS. In comparison to men, women exhibit higher HRV and a greater baseline vagal tone [68]. Paradoxically, women also have slightly faster heart rate, averaging 3–5 beats per minute higher than men [69]. Burke et al. conducted a study on gender differences in heart rate before and after autonomic blockade, which demonstrated that these disparities in heart rate are not solely attributed to autonomic nerve regulation [70]. Furthermore, women tend to have shorter QRS duration, lower QRS voltage, and flatter ST segment [69,71]. Additionally, being female is also an independent risk factor for long-QT-dependent cardiac arrhythmias [72,73].

The aforementioned phenomenon demonstrates that autonomic neurology alone is insufficient to fully elucidate the mechanisms underlying arrhythmia and depressive disorder. It is crucial to consider other factors interacting with the ANS, such as the structural and functional characteristics of the female heart (e.g., smaller size and thinner ventricular walls), which affect electrical conduction and excitability [68,74]. Additionally, the diverse effects of different sex hormones on heart rate and mood [68,73,75], and the lower adrenergic response to mental stress in women [68,76], should be considered. Therefore, the pivotal roles of physiological systems, such as the endocrine and immune systems, must be acknowledged in governing the intricate interplay between the heart and brain [35].

## 4. Relationship of Interactions

**Figure 2:**
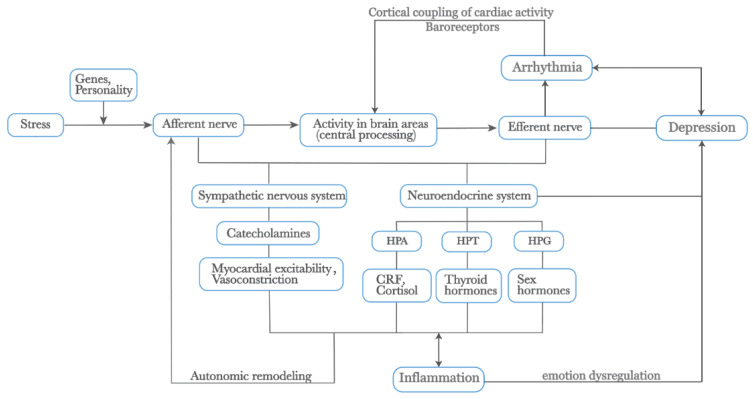
External stress mediates depression and arrhythmia by affecting physiological changes within the body. Additionally, the activation of the SNS releases catecholamines, increasing myocardial excitability and vasoconstriction. Prolonged physiological changes lead to autonomic remodeling. The neuroendocrine system, closely linked with the ANS, including the HPA, HPT, and HPG axes, releases CRF, cortisol, thyroid hormones, and sex hormones, all of which play roles in the heart–brain relationship. The SNS and HPA axis also promote inflammatory responses, which interact to mediate emotional dysregulation. Furthermore, the cortical coupling of cardiac activity through baroreceptors is crucial in these processes. Genes and personality traits also play significant roles in this pathway. Abbreviations: SNS: Sympathetic Nervous System, ANS: Autonomic Nervous System, HPA: Hypothalamic-Pituitary-Adrenal, HPT: Hypothalamic-Pituitary-Thyroid, HPG: Hypothalamic-Pituitary-Gonadal, CRF: Corticotropin-Releasing Factor.

The following section elucidates additional factors influencing the interplay between arrhythmia and depression based on autonomic anatomy (See Figure 2).

### 4.1. Genes

Genes play a pivotal role in the structure and functioning of both the heart and brain [77,78,79,80]. Twin studies suggest that MDD has a heritability rate ranging from 30% to 50%, with offspring of at least one affected parent having a 2 to 3-fold increased risk compared to unexposed offspring [81,82,83]. A recent Mendelian randomization study involving 40,000 participants revealed a genetic correlation between cardiac traits and psychiatric disorders. For instance, a genetic association was observed between left atrial characteristics and neuroticism, and between left ventricular traits and schizophrenia and bipolar disorder. Additionally, cognitive features and neuroticism were genetically linked to right heart (atrial and ventricular) characteristics [84]. Genetic differences and hereditary variations regulate physiological responses to external stress and influence susceptibility to certain diseases. Genetic differences associated with dopamine and serotonin release play a role in mediating fear-induced bradycardia in the human brain [85]. Individuals carrying one or two copies of the short allele of serotonin transporter (SERT) promoter polymorphism are more susceptible to stressful events and depression [86], whereas a single amino acid (G406R) mutation could lead to long QT syndrome type 8 [87].

### 4.2. Personality Traits

Personality traits are closely associated with depressive disorders [88], and can serve as both symptoms and potential precursors or causative factors of depression [89]. Certain personality characteristics increase vulnerability to arrhythmia and depression [90]. For instance, ruminative response style has the prospective ability to predict the onset of depression [89]. Worry and rumination tendencies are independent risk factors for CVD, prolonging stress-induced emotional and physiological activation, thus mediating the relationship between depression/anxiety and CVD risk [90]. Additionally, the Type D personality has been demonstrated as an independent factor associated with compromised patient-reported physical and mental health status across diverse cardiovascular patient groups [91].

### 4.3. Stress

Stress, triggered by an imbalance in homeostasis or external unpleasant challenges [92], is a potent risk factor for depression [93]. Physiological changes induced by stress are believed to mediate the onset and progression of both depression and CVD [94,95,96,97]. From a physiological perspective, acute mental stress events result in increased release of catecholamines and glucocorticoids, and activation of the SNS and HPA axis [16,96]. Over-activation and dysregulation of stress systems can disrupt neural remodeling and psychological resilience, contributing to a detrimental cycle of emotional disorders [95]. Chronic mental stress frequently activates and prolongs the stress response, leading to the reconstruction of physiological and behavioral responses, and promoting structural and functional changes in brain regions similar to those observed in depression [95,96,98]. Even stressful events occurring during childhood can lead to persistent sensitization of CNS circuit along with heightened pituitary-adrenal and autonomic responses during adulthood [99,100]. Microscopically, stress-induced mitochondrial dysfunction enhances reactive oxygen species production, playing a crucial role in the pathogenesis of diseases including CVD and depression [97,101].

### 4.4. Endocrine System

The hypothalamus plays a crucial role in regulating various physiological activities essential for survival and maintaining homeostasis, including energy metabolism, reproduction, water and electrolyte balance, body temperature regulation, sleep-wake cycles, and stress response [102]. It is also involved in descending regulation related to emotion, such as HPA axis, hypothalamic-pituitary-thyroid (HPT) axis, hypothalamic-pituitary-gonadal (HPG) axis, and other endocrine reactions [35]. For instance, excessive secretion of corticotropin-releasing factor due to stress can lead to exaggerated activation of the pituitary-adrenal axis and ANS [95,96], contributing to the association between depression, hypercortisolemia, and arrhythmia [16,103]. Hypothyroidism is often associated with depressive symptoms, and a significant proportion of patients with depression exhibit thyroid dysfunction [75,104].

The hypothalamus plays a crucial role in ascending regulation by receiving visceral information transmitted through the NTS and PBN [34]. Alterations in cardiac activity impact the brain and mood via ascending pathways [34], such as arrhythmias and stress responses, which can induce changes in the ANS, cardiovascular volume, glucocorticoid levels, mineralocorticoids, and other endocrine components. Glucocorticoid receptors and mineralocorticoid receptors are widely distributed in various organs, including the brain and heart [95], with high expression in the hippocampus, a critical region for learning and memory [96,105]. Adrenal steroids significantly influence hippocampal neuronal plasticity and loss [95,96,105]. Cumulative exposure to high cortisol levels is associated with hippocampal atrophy, leading to cognitive impairment [96,106]. In addition to the HPA axis, primary abnormalities of the HPT and HPG axis are often associated with alterations in cardiac rhythm and mood. Drugs targeting endocrine function often affect mood and cardiac rhythm; for instance, T3 can be used as an adjunct to antidepressants when conventional medications are ineffective [46,75].

The effects of sex hormones on arrhythmia susceptibility encompass several mechanisms: (1) Sex hormones induce functional physiological changes by modulating transcriptional regulation; (2) Sex hormones activate transcription factors via the mitogen-activated protein kinase (MAPK) pathway; (3) Cardiac ion channel activity is regulated by sex hormones through endothelial nitric oxide synthase (eNOS) activation; and (4) Rapid modulation of cardiac ion channel activity by sex hormones occurs directly through the PI3K/Akt/eNOS pathway; as well as other potential pathways [68,73]. Testosterone primarily increases K^+^ current (IKs) and inhibits L-type Ca^2+^ current (ICa,L) to shorten APD and QT interval, exhibiting a dose-dependent effect [68,73]. There is a significant gender disparity in the prevalence of depressive disorders, with women being approximately 1.7 times more likely to experience depression compared to men [107]. However, there is minimal divergence in the proportion of affected men and women before puberty [75]. The incidence of depression begins to diverge as estrogen levels rise significantly, with depression being almost twice as common in women as in men during their reproductive years. Fluctuations in sex hormones among women are associated with an elevated risk of mood disorders such as depression [75]. It is believed that the organizational effects of gonadal hormones during early development contribute to sex differences in brain structure, potentially explaining the heightened vulnerability of females to depression; however, the precise mechanism remains unclear [108].

**Figure 3:**
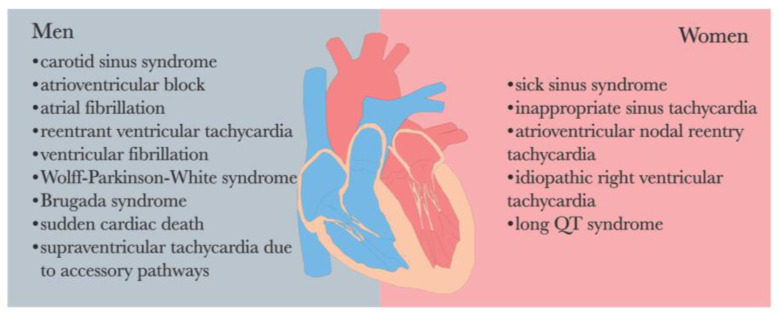
Sex differences in the prevalence of cardiac arrhythmias [69,109].

The endocrine system and ANS are intricately interconnected in the regulatory processes of the human body. It is worth noting that a significant gender disparity exists in the prevalence of arrhythmia [69,109] (See Figure 3). Perturbations in endocrine function and hormonal activity may play a pivotal role in the pathological mechanisms underlying various diseases, including depression and arrhythmia [46].

### 4.5. Inflammation

Psychological stress can activate the HPA axis and SNS, promoting an inflammatory response [3,98,110,111]. The HPA axis and SNS stimulate the release of cortisol and catecholamines, which regulate inflammation by exerting immunosuppressive effects and inhibiting white blood cell activation and inflammatory cytokine production [112,113]. There is a bidirectional relationship between inflammation and the ANS. Inflammatory factors can activate the HPA axis and SNS [112], leading to increased myocardial electrical instability. Elevated levels of pro-inflammatory cytokines accelerate endothelial dysfunction progression, atherosclerosis development, arrhythmia occurrence, etc., even remodeling cardiac function [3,97].

Inflammation can impact brain structures involved in emotion regulation, such as the hippocampus, amygdala, and prefrontal cortex, and may indirectly influence the mood regulation network through various pathways [114]. Dysfunctional emotion regulation and high-stress susceptibility can lead to abnormal brain responses to emotional stimuli, triggering aberrant neuroendocrine effects, including heightened HPA axis and SNS activity, ultimately contributing to elevated central and peripheral inflammation [114]. The chronic proinflammatory state may exacerbate disorders related to emotional regulation [114]. There is a bidirectional positive relationship between inflammation and emotion dysregulation [114]. Studies have shown that the development of depressive symptoms is associated with elevated levels of proinflammatory cytokines, regardless of depression diagnosis [110,115]. Increased inflammation is linked to greater depression severity and reduced responsiveness to antidepressant treatment, suggesting that inflammation might contribute to treatment resistance [112]. In patients with depression, levels of interleukin (IL)-6 and tumor necrosis factor (TNF) are significantly increased in blood and cerebrospinal fluid [110,111]. IL-6 is positively correlated with the duration of the current depressive episode and total antidepressant treatment duration, serving as an indicator of acute deterioration [116]. TNF-α levels correlate with the duration of the current episode [116]. Elevated C-reactive protein levels are significantly associated with somatic symptoms of depression [47]. In recent years, indirect inflammatory markers such as the neutrophil-lymphocyte ratio (NLR) have gained prominence as biomarkers for assessing overall inflammatory status. Studies have demonstrated a positive association between NLR and depressive symptoms, with this relationship exhibiting gender specificity: NLR is positively correlated with depressive symptoms in women but not in men [117]. Different types of female depression also show variations in chronic immune-inflammatory markers. Specifically, elevated mid-trimester NLR is independently associated with antenatal perinatal depression rather than postpartum depression (PPD) [117]. Recent investigations have indicated no correlation between indirect inflammatory markers like the monocyte-lymphocyte ratio and PPD [118]. Despite these findings, the exact etiology of depression remains elusive. Consequently, there has been growing interest in exploring the potential link between depression and the immune system [117]. These results highlight the necessity for further research into the complex relationship between inflammation and various subtypes of depression, providing valuable insights into their association.

### 4.6. 5-Hydroxytryptamine

The involvement of 5-hydroxytryptamine (5-HT) in the pathogenesis of depression and CVD is crucial [24]. Individuals with 5-HT system disorders are more susceptible to depression [24,119]. 5-HT regulates the contractile function of the human heart through interaction with the 5-HT_4_ receptor [120]. Arrhythmia increases the risk of stroke [121], and post-stroke malignant ventricular arrhythmia can even result in sudden cardiac death [122]. Furthermore, approximately one-third of stroke survivors experience clinically significant symptoms of depression within 12 months [123].

Recent studies have identified hyperacute dysregulation of the 5-HT axis in brain ischemia [124], which may be a key mechanism underlying post-stroke depression. Alterations in platelet SERT and 5-HT_2A_ receptor (5-HT_2A_R) densities may increase susceptibility to ischemia or indicate the presence of a thrombotic process [124]. Reduced platelet SERT density has been observed in conjunction with depression [124,125], while certain antidepressants, such as clomipramine, lead to increased platelet 5-HT_2A_R density and aggregation response [124,125]. Although the specific association between 5-HT and depression remains inconclusive [126], these findings suggest that abnormalities in platelet SERT and 5-HT_2A_R could be potential links between depression and brain ischemia [124]. Additionally, selective serotonin reuptake inhibitors, commonly used as antidepressants, can reduce morbidity and mortality associated with CVD by effectively treating depressive symptoms alongside serotonin and platelet abnormalities [24]. In conclusion, understanding the role of 5-HT in both depression and CVD is essential [24,120].

### 4.7. Behavioral Factors

Depression has been demonstrated to exert a detrimental impact on neurocognitive functions as well as instrumental activities of daily living [96,127,128]. One of the core symptoms of depression is sleep disturbance [129], which can lead to cognitive impairment [96] and negatively affect financial capacity [130], potentially contributing to the onset and progression of depression. Physiologically, lack of sleep can result in insulin resistance, elevated nocturnal cortisol levels, reduced brain glucose utilization rate, increased ghrelin secretion, decreased leptin production, heightened SNS activity and other ANS imbalances, as well as endocrine disorders and other physiological changes [131,132,133,134]. Among these effects, heightened ghrelin levels coupled with decreased leptin levels may contribute to increased appetite. Additionally, impaired glucose regulation can impact hippocampal volume and function [96,105]. These physiological adaptations are implicated in the pathogenesis of heart disease, obesity, depression as well as the promotion of unhealthy lifestyles [135,136,137,138]. A study revealed that individuals who experience insufficient sleep are at a higher risk for hypertension (17% increase), CVD (16% increase), coronary heart disease (CHD) (26% increase), and obesity (38% increase) [137]. Furthermore, patients with mental disorders exhibit lower adherence to healthy lifestyle practices [138]. Unhealthy behaviors such as smoking, excessive alcohol consumption, and physical inactivity exacerbate adverse physiological changes, contributing to arrhythmia development and worsening depressive symptoms [96,135,136,137,138,139].

Poor lifestyle behaviors can lead to anemia, which is a significant global public health issue that affects physical and mental abilities [140]. Anemia not only alters heart rate but also increases the risk of PPD for pregnant women during and after pregnancy [141,142,143]. However, one study found no negative impact of maternal prepartum anemia on the likelihood of developing postpartum depressive symptoms within the first 3 days after delivery [140]. Further research is needed to explore the relationship between anemia in women and symptoms of PPD. PPD is a major risk factor for suicide in postpartum mothers, bringing mental stress to families, affecting family relationships, and adversely impacting the emotional and cognitive development of offspring [140].

## 5. Discussion

Depression and arrhythmia are both prevalent and serious global health issues. This study aims to investigate the bidirectional relationship between depression and arrhythmias and elucidate their underlying mechanisms. Understanding the interaction between these conditions will not only shed light on the pathogenesis of mood disorders and cardiac diseases but also offer new insights for clinical treatment strategies.

In this narrative review, we propose that the ANS serves as the anatomical foundation linking the heart and brain, constituting a shared pathophysiological mechanism underlying both depression and arrhythmias. The heart and brain engage in bidirectional communication via interoceptive pathways. The information exchange within the HBA is both bidirectional and complex, influenced by inherent genetic factors and variable physiological responses to external stimuli. This dynamic interplay plays a crucial role in the development and comorbidity of mood disorders and CVD.

Numerous studies have consistently demonstrated a correlation between depression and an elevated heart rate, as well as reduced HRV [57,58,59]. Imbalances in the ANS are recognized as critical mechanisms underlying arrhythmia [52]. Disruptions in the ANS may serve as a shared pathological mechanism contributing to both arrhythmias and various mood disorders [52,54,55,56,57]. Research has elucidated that the interplay between autonomic nerves, hormones, and cytokines forms the physiological connection within the HBA [27]. At a biological level, stressful events can trigger the activation of both the SNS and the HPA axis [16,96], thereby facilitating the onset and progression of both depression and arrhythmia. These findings further substantiate a dual pathological mechanism involving ANS dysfunction in these two conditions. Additionally, it is noteworthy that SNS and HPA axis activation also promotes an inflammatory response [3,98,110,111], with increased levels of proinflammatory cytokines being associated with disease progression in both depression and arrhythmia [3,97,110,115]. Furthermore, unhealthy lifestyles resulting from these conditions can establish a detrimental cycle perpetuating both arrhythmia and depression [96,135,136,137,138,139]. Innate genetic factors and personality traits also significantly influence disease susceptibility for both conditions [84,90].

This article discusses the neural basis of the ANS and its association with the quality and intensity of emotional experiences [33]. Previous studies have confirmed the role of ascending pathways to some extent. Specifically, β-adrenergic blockers, which reduce SNS activity, may mitigate negative, high-arousal emotions by attenuating peripheral signals [144]. Patients with spinal cord injury, characterized by disrupted nerve communication from the periphery to the brain, often struggle to assess their emotional responses to complex scenarios that elicit fear and anger [145].

This review holds significant scientific and clinical relevance. Exploring the interaction between depression and arrhythmia helps elucidate the common pathological mechanisms underlying emotional disorders and CVD, which may reduce morbidity and mortality associated with both conditions and improve quality of life. Building on previous work, this study summarizes the bidirectional relationship between depression and arrhythmia, emphasizing the critical role of the ANS in this process. It examines the impact of emotional disorders on cardiovascular function and how heart activity, through interoceptive pathways, influences brain function and emotional states. By comprehensively analyzing the existing literature, this study elucidates the mechanisms of the bidirectional effects of ANS dysfunction on depression and arrhythmia and the interaction of other factors influencing these conditions.

## 6. Current Clinical Status

Interventions for patients with arrhythmias include pharmacotherapy, catheter ablation, implantable devices, surgical procedures, and lifestyle modifications [146]. Depression is significantly associated with an increased incidence and risk of new-onset AF [147]. Nearly 20% of implantable cardioverter-defibrillator recipients suffer from anxiety and depression, which are linked to increased mortality, necessitating psychological interventions for these patients [148]. Depression triples the risk of medication nonadherence and noncompliance with medical regimens [149] and exerts a pathological impact on the heart. Most studies indicate a dose-response relationship between depression severity and cardiac events, with more severe depression correlating with earlier and more serious cardiac incidents [149]. The American Heart Association’s treatment guidelines recommend evaluating depressive symptoms in cardiac patients and incorporating depression management into routine cardiac care [149].

Current treatment strategies for the comorbidity of arrhythmias and depression include both pharmacological and non-pharmacological approaches [24]. Adding antidepressants to the medication regimen of cardiac patients has shown some efficacy; however, antidepressants may reduce vagal control of the heart [62,150,151,152,153,154,155,156,157], and can cause arrhythmias, autonomic dysfunction, endocrine disturbances, and sleep disorders [158]. Common antiarrhythmic drugs, such as beta-blockers, may lead to bradycardia and depression [148]. Non-pharmacological treatments include cognitive-behavioral therapy, aerobic exercise, and cardiac rehabilitation [149]. Psychotherapy is a safe treatment option for patients who cannot tolerate antidepressants and can also benefit cardiac patients with depression [24,148]. Aerobic exercise and cardiac rehabilitation improve cardiovascular health and reduce depressive symptoms [149,159,160], but exercise prescriptions must be individually tailored based on the patient’s cardiac condition and exercise capacity [149]. Increasingly, research is adopting a transdiagnostic approach to understand and treat mental health issues, moving beyond traditional diagnostic categories [161]. Interoception is considered a common factor across various mental health conditions, including anxiety, depression, and other mental disorders [162]. Studies have demonstrated the effectiveness of interoceptive-based psychological interventions in managing these issues [162].

In interoception research, the heart is the most commonly studied visceral organ, and the heartbeat has been the most extensively examined interoceptive sensation [33,163]. Recent studies have shown that patients with AF who underwent catheter ablation experienced more significant improvements in depressive symptoms compared to those who received only antidepressant medication [19]. Brian Hsueh et al. found that optically evoked tachycardia can effectively enhance anxiety-like behavior in mice [164], which may partially support the role of interoception in emotion regulation.

Research on interoception has increased sixfold over the past decade, achieving significant progress in understanding its neurobiology and its connection to emotional experiences [33]. However, the current concept of the interoceptive pathway remains broad and somewhat ambiguous [33,165]. Further research into chemical and neural signals from various systems, such as the immune system, endocrine system, and somatosensory tissues, may advance a more detailed understanding of the interoceptive pathway [33]. Emphasizing interdisciplinary collaboration can facilitate deeper insights into these pathways. Recent studies utilizing advanced signal processing techniques and synthetic data generation models based on electrocardiogram (ECG) and EEG data offer a comprehensive and precise analysis of heart–brain interactions during emotional arousal [166].

## 7. Limitation

There are many challenges in understanding the specific roles of heart–brain interactions. Although strong evidence indicates that emotional disorders are characterized by changes in ANS activity [54,66], it remains unclear whether ANS dysfunction is a cause or a consequence of depression [167]. Additionally, there is a lack of research specifically linking certain emotional activities with changes in ANS activity [54,66]. Depression and CVD often co-occur, with most research focusing on the relationship between MDD and CHD [61,168,169]. However, studies on the connection between arrhythmias and mood disorders are relatively scarce. Most existing studies on the heart–brain relationship are cross-sectional, lacking longitudinal follow-up data that could elucidate the temporal sequence of these conditions [3]. In addition, previous research has shown that psychological stress can lead to ventricular arrhythmias [16,18], but mood disorders also play a significant role in non-ventricular arrhythmias [19,20,21,170]. ECG studies have found close associations between P-wave parameters and adverse cardiovascular and neurocognitive outcomes [171]. However, clinical studies focusing on atrial signals such as the P-wave and their relationship with emotions are currently very rare. It is noteworthy that there are significant gender differences in both arrhythmias and mood disorders [72,73,107], which is an important factor in the bidirectional relationship between depression and arrhythmias. This disparity is reflected in cardiac anatomy, electrophysiology, and treatment strategies. Female participants are underrepresented in arrhythmia-related studies, often leading to extrapolation from predominantly male samples to female populations [68,172]. Although previous studies have partially confirmed the ascending interoceptive pathways, other research using human intracranial electrophysiology, an invasive technique, suggests that emotional states originate from complex brain networks [173]. Findings on the causal relationship between interoception and emotions are inconsistent, and current research lacks sufficient conditions to establish causality firmly [33]. In summary, while there is strong evidence linking emotional disorders with changes in ANS activity, more research is needed to clarify the directionality of this relationship and to better understand the specific mechanisms involved, particularly considering gender differences and the complexity of interoceptive pathways.

Additionally, this review is a narrative review, which has methodological limitations compared to systematic reviews. We only searched for literature using English keywords in the PubMed and Web of Science databases, which may have resulted in the omission of important non-English studies or research not indexed in these databases. The selected literature may also be subject to publication bias, where studies with positive results or significant findings are more likely to be published, while studies with negative results or no significant findings may not be included. This review is based on our analysis and summary of all the cited literature. While we aimed to be as objective as possible in our analysis, the process inevitably involves subjective summarization.

## 8. Future Perspectives

In future research, several important directions can be pursued to further elucidate the intricate relationship between depression and arrhythmias. Firstly, more longitudinal studies are needed to better understand the causal relationship between depression and arrhythmias and their long-term effects. Longitudinal research can help determine the temporal sequence of these conditions and uncover their underlying pathophysiological mechanisms. Moreover, increasing sample sizes, particularly by including more female participants, will aid in elucidating the impact of gender on the interaction between depression and arrhythmias. Investigating gender differences in these conditions will provide more comprehensive clinical guidance and reveal the specific roles that gender plays in arrhythmias and mood disorders.

Furthermore, more interdisciplinary research is needed, combining approaches from neuroscience, cardiovascular medicine, psychology, and other related fields to thoroughly explore the bidirectional role of ANS dysfunction in depression and arrhythmias. Technological applications are crucial in this regard. Previous studies have used magnetic resonance imaging (MRI), functional MRI, and PET imaging techniques to observe changes in brain structure and function, assessing cerebral metabolic activity. Techniques like ECG, EEG, and HRV overcome the limitations of low temporal resolution, allowing real-time monitoring of cardiac and brain electrical activity and analyzing the relationship between heartbeat activity and brain function [16,166]. Peripheral physiological markers reflecting ANS activity, such as electrodermal activity, blood pressure variability, pupillary light reflex, respiratory rate and patterns, and skin temperature, are also crucial for understanding the pathophysiology of depression [174,175,176]. Additionally, research is exploring the application of repetitive transcranial magnetic stimulation (rTMS) in heart–brain interactions, using peripheral biomarkers to gain insights into the effects of rTMS on ANS function [167,175,177]. This approach may offer optimized treatment strategies for depression. Compared to the confounding effects of antidepressants on the ANS, rTMS could be a better technique for investigating heart–brain relationships [167]. However, non-invasive techniques may limit the manipulation and measurement of peripheral processes. New methods, such as deep brain stimulation or vagus nerve stimulation, offer promising directions for future research by allowing the manipulation and measurement of tissue function [33].

## 9. Conclusions

This review summarizes existing studies demonstrating the complex bidirectional relationship between depression and arrhythmia. Dysfunction of the ANS is a significant pathological mechanism underlying both conditions. Additionally, the interoceptive pathway based on the ANS provides a novel perspective for understanding heart–brain interactions. However, multidisciplinary perspectives should be considered in the study of the HBA to address gaps in our understanding of heart–brain interactions and emotion generation.

Timely identification and intervention of comorbidities are crucial in clinical practice. Screening patients with heart disease for depressive symptoms, as well as assessing cardiovascular health in patients with depression, are essential steps. Currently, the clinical diagnosis and treatment of mental disorders tend to transcend traditional diagnostic categories. Our study highlights the importance of viewing the body as a complex, interconnected system rather than treating organs in isolation. Future research should further investigate the interaction of multiple risk factors and promote interdisciplinary research to uncover deeper mechanisms. By deepening our understanding of these mechanisms, we can anticipate significant advances in improving patient quality of life and disease management.
